# PD1-IL2v expands and induces effector CD8^+^ TILs, but not Tregs, in the BCG treated orthotopic non-muscle invasive bladder cancer model

**DOI:** 10.1186/s13046-026-03667-w

**Published:** 2026-02-11

**Authors:** Irene Locatelli, Marco Lorenzoni, Chiara Venegoni, Alessia Di Coste, Rita Sorrentino, Jithin Jose, Daniela Maria Cirillo, Patrick Weber, Amrita Manchala, Ralf J. Hosse, Valeria Nicolini, Pablo Umana, Christian Klein, Andrea Salonia, Francesco Montorsi, Matteo Bellone, Marco Moschini, Laura Codarri Deak, Massimo Alfano

**Affiliations:** 1 Comprehensive Cancer Center, Unit of Urology, URI, IRCCS Ospedale San Raffaele, Milan, Italy; 2 Department of Immunology, Transplantation and Infectious Diseases, Cellular Immunology Unit, IRCCS Ospedale San Raffaele, Milan, Italy; 3 Division of Immunology, Transplantation and Infectious Diseases, Emerging Bacterial Pathogens Unit, IRCCS Ospedale San Raffaele, Milan, Italy; 4 FUJIFILM Visualsonics Inc, Amsterdam, The Netherlands; 5 Roche Innovation Center Zurich, Schlieren, Switzerland; 6 Department of Biochemistry, Faculty of Chemistry and Pharmacy, Ludwig Maximilians University of Munich, Munich, Germany; 7 Vita-Salute San Raffaele University, Milan, Italy

**Keywords:** Bladder cancer, BCG, ICI, PD-1, PD1-IL2v, CD8, Tregs

## Abstract

**Background:**

The combination of systemic immune checkpoint inhibitors with standard of care intravesical Bacillus Calmette-Guerin (BCG) therapy for non-muscle invasive bladder cancer (NMIBC) has shown potential in enhancing BCG therapeutic effects. However, challenges such as disease recurrence, progression, and immune-related toxicities remain a significant hurdle. Furthermore, the mechanism of action of BCG involves broad, non-specific immune activation. While this recruits bystander CD8^+^ and NK cells, it also triggers the undesired expansion of Tregs, which may ultimately hamper therapeutic efficacy. We aimed to explore the feasibility of a targeted approach designed to overcome intravesical tumor resistance and selectively enhance the expansion and effector functions of CD8^+^ TILs.

**Methods:**

We tested intravesical administration of BCG with murinized PD1-IL2v, a fusion protein that simultaneously targets PD-1 and IL-2Rβγ in cis on the same cell, in a preclinical mouse model of NMIBC.

**Results:**

Both BCG monotherapy and co-treatment with a murinized PD1-IL2v improved animal survival. Notably, the intravesical combination of BCG and murinized PD1-IL2v significantly increased the number of CD8^+^ TILs with polyfunctional cytotoxic effector phenotype and avoided Treg expansion in contrast to BCG monotherapy. In addition, in patient-derived tumors, we observed a higher frequency of CD8⁺ TILs (18%) compared to Tregs (5%), with 60% of the CD8^+^ TILs expressing PD-1 on their surface, representing a potential target for PD1-IL2v, alias eciskafusp alfa.

**Conclusions:**

The intravesical combination of BCG with murinized PD1-IL2v selectively increases the number of cytotoxic CD8^+^ TILs, but not Tregs, and improves the survival of the mice in the preclinical model of bladder cancer model. These findings support the rationale for exploring the clinical use of eciskafusp alfa for intravesical administration in high-risk NMIBC patients. The combination of BCG and eciskafusp alfa could be deployable as a second line of treatment for NMIBC patients unresponsive to BCG.

**Supplementary Information:**

The online version contains supplementary material available at 10.1186/s13046-026-03667-w.

## Introduction

Intravesical Bacillus Calmette-Guerin (BCG) therapy is the gold standard adjuvant treatment for intermediate- and high-risk non muscle invasive bladder cancer (NMIBC) [1]. However, despite cycles of weekly instillations and frequent control cystoscopies, recurrence and progression of the disease occur in 80% and 45% of patients, respectively [2]. Low efficacy of adjuvant therapies together with frequent bladder cancer (BCa) recurrence and related reduction of patient’s quality of life contribute to making BCa the most expensive neoplasm in terms of healthcare spending [3].

As with other malignancies, immune checkpoint inhibitors (ICIs) have emerged as a treatment option also for NMIBC [4–7]. Systemic administration of ICIs in combination with intravesical BCG has shown promise in several phase 1b/2 studies [8, 9], diversifying how ICIs can be used. Despite promising clinical results, concerns remain about these treatment strategies, mainly related to immune-related adverse events, toxicity and resistance [6, 7, 10, 11]. One hypothesis to explain drawbacks in the application of anti-PD-1/PD-L1 therapy in BCa is associated with the lack of an efficient local antitumor response, while systemic inhibition of the immune-checkpoint PD-1 is responsible for the immune-related toxicity. ICI related adverse events can be reduced in case of intravesical instillation, as supported by PemBla Phase 1 study that reported no systemic immune effects in six patients that received six weekly intravesical instillations of pembrolizumab [12].

Therefore, immunomodulatory strategies that disrupt the underlying intravesical non-reactivity are needed. One approach consists in increasing the number of cytotoxic CD8^+^ TILs. This can be done by providing an agonist signal that preferentially activates IL-2 pathway in cis on PD-1^+^ T cells, meaning on the same cell, through the IL-2Rβγ heterodimeric receptor without concomitantly activating IL-2Rα (CD25), and consequentially amplifying Tregs, while blocking the PD-1 receptor. This strategy can be achieved using the engineered immunocytokine PD1-IL2v, alias eciskafusp alfa [13]. Herein, we evaluated the effects of the intravesical administration of murinized PD1-IL2v in a mouse model of orthotopic NMIBC in human PD-1 transgenic mice. We monitored for survival of tumor bearing mice and characterized the immunophenotype of tumor-infiltrating lymphocytes of animals treated with BCG monotherapy and in combination with murinized PD1-IL2v.

As a benchmark for in vivo efficacy, we used murinized anktiva (nogapendekin alfa inbakicept)-like IL-15 superagonist complex (N-803), composed by a dimeric IL-15 with N72D mutation and a dimeric IL-15Rα sushi domain fused to the Fc portion of a murine IgG2a Fc fusion protein, with increased affinity for the beta subunit of the IL-2R. Anktiva mechanism of action has been reported to promote nonspecific proliferation, activation and effector functions of NK cells and bystander CD8^+^ T cells, independently of PD-1 expression [14], in a manner similar to that of BCG mechanism of action. In clinical studies, when administered in combination with BCG, anktiva led to grade 1–2 adverse events in more than 86% of patients and demonstrated a favorable prognosis in BCG-unresponsive NMIBC patients [15, 16].

## Materials and methods

### Cis-trans STAT5-P assay on PD-1^+^ and PD-1^−^ T cells with Eciskafusp Alfa and anktiva-like molecule

CD4^+^ T cells from healthy donor PBMCs were sorted with CD4 beads (130-045-101, Miltenyi) and activated for 3 days in presence of 1 µg/ml plate-bound anti-CD3 (overnight pre-coated, clone OKT3, #317315, BioLegend) and 1 µg/ml of soluble anti-CD28 (clone CD28.2, #302923, BioLegend) antibodies to induce PD-1 expression. Three days later, the cells were harvested and washed several times to remove endogenous cytokines and half of the cells were labeled with Cell Trace Violet (CTV) (5 µM, 5 min at room temperature (RT); C34557, Thermo Scientific) and the other half were left unlabeled. Then, the unlabeled cells were incubated with a saturating concentration of a competing anti-PD-1 antibody (in-house molecule, 10 µg/ml) for 30 min at RT followed by several washing steps to remove the excess unbound anti-PD-1 antibody. Thereafter, the PD-1 pre-blocked unlabeled cells (25 µl, 6*10^6^ cells/ml) were co-cultured 1:1 with the PD-1^+^ CTV-labeled cells (25 µl, 6 × 10^6^ cells/ml) in a V-bottom plate before being treated for 12 min at 37 °C with increasing concentrations of treatment immunoconjugates (50 µl, 1:10 dilution steps). To preserve the phosphorylation state, an equal amount of Phosphoflow Fix Buffer I (100 µl, 557870, BD Bioscience) was added after 12 min incubation with the various constructs to allow the IL-2R signaling upon binding to PD-1. The cells were then incubated for an additional 30 min at 37 °C for fixation before being permeabilized overnight at -80 °C with Phosphoflow PermBuffer III (558050, BD Bioscience). On the next day, STAT-5 in its phosphorylated form was stained for 30 min at 4 °C by using an anti-STAT-5P antibody (47/Stat5(pY694) clone, 562076, BD Bioscience). The cells were acquired at the flow cytometer (FACS) BD-Symphony A5 (BD Bioscience) instrument. The frequency of STAT-5P was determined with FlowJo (V10) and plotted with GraphPad Prism.

### Functional activity of Eciskafusp Alfa and anktiva-like molecule on cytotoxic effector functions and proliferation of allo-specific PD-1^+^ CD4^+^ T cells

CD4 isolation and CTV labelling was conducted as described above. The sorted CD4^+^ T cells were co-cultured with irradiated (50 Gy) 647-V (human urothelial bladder cancer cell line) in an E: T ratio of 5:1 (100,000 T Cells: 20,000 647-V) in the presence or absence of increasing doses of eciskafusp alfa or anktiva-like molecule. The cells were co-cultured in a 96-round bottom plate for 5 days at 37 °C, 5% CO_2_. After 5 days, the accumulation of cytokines in the Golgi complex was enhanced by applying Protein Transport Inhibitors (GolgiPlug™, 555029, BD Bioscience; and GolgiStop™, 554724, BD Bioscience) for 5 h prior to the FACS staining. The cells were first stained for CD4 and for live/dead. After overnight fixation/permeabilization (554714, BD Bioscience), the cells were stained intracellularly for Granzyme B (GrzB). The cells were acquired at the FACS BD-Symphony A5 (BD Bioscience) instrument and analyzed with FlowJo^®^ and GraphPad Prism^®^. By gating on the living and proliferating CD4^+^ T cells (CTV^low^), the frequency and mean fluorescence intensity of GrzB secretion was compared between the conditions.

### PD-1 staining of tumor-infiltrating lymphocytes from patients with bladder cancer

Frozen single-cell suspensions of human PBMCs and TILs obtained from bladder cancer patients were purchased from Discovery Life Sciences (Huntsville, AL). After thawing and washing steps, the cells were incubated at 37 °C for 3–4 h to allow re-expression of thermosensitive surface markers. The cells were then stained with the surface antibodies listed in Supplementary Table S1 at 4 °C for 30 min before being fixed and permeabilized overnight with the BD transcription factor buffer (Transcription factor buffer set, 562574; BD Pharmingen; Franklin Lakes, NJ). After washing with the BD permeabilization buffer, the cells were incubated at room temperature for 1 h in the presence of a rabbit anti-PD-1 antibody, which recognizes an intracellular domain of PD-1 not competing with anti-PD-1 antibodies binding to the surface. After washing, cells were incubated with a secondary anti-rabbit-BV421 antibody for 30 min. Finally, the cells were stained with anti-TCF-1 and FOXP3 antibodies at RT for 1 h before a final washing step to remove the unbound antibody.

### Assessing preferential binding of Eciskafusp Alfa on PBMCs and TILs

Frozen single cell suspensions of human PBMCs and TILs obtained from bladder cancer patients were purchased from Discovery Life Sciences (Huntsville, AL). After thawing and washing steps, the cells were incubated at 37 °C for 3–4 h to allow re-expression of thermosensitive surface markers. Ex-vivo binding of eciskafusp alfa and FAP-IL2v was performed by exposing the plated PBMCs and TILs to 630 pM of both constructs for 30 min at RT. After a washing step to remove unbound molecules, cells were incubated for an additional 30 min at 4 °C with a PE-labelled antibody recognizing the P329G LALA mutation in the Fc portion of the primary. Following another washing step, cells were stained for 30 min at 4 °C with the following antibodies to characterize the phenotype of the immune-cytokine targeted cells: anti-CD3 (1:100; clone OKT3, BioLegend), anti-CD45 (1:100, clone HI30, Biolegend), anti-CD4 (1:100; clone OKT4, BD Biosciences) and anti-CD8 (1:100; clone RPA-T8, BD Biosciences). eBioscience™ Fixable Viability Dye eFluor™ 780 (1:1000, Thermofisher). The cells were then fixed and permeabilized overnight with BD transcription factor buffer (Transcription factor buffer set, 562574; BD Pharmingen; Franklin Lakes, NJ). After washing with the BD permeabilization buffer, the cells were incubated at RT for 1 h in the presence of a rabbit anti-PD-1 antibody, which recognizes an intracellular domain of PD-1 not competing with anti-PD-1 antibodies binding to the surface. After washing, cells were incubated with a secondary anti-rabbit-BV421 antibody for 30 min. Finally, the cells were stained with anti-TCF-1 and FOXP3 antibodies at RT for 1 h before a final washing step to remove the unbound antibody. Sample acquisition was performed on a BD Symphony A5 instrument with FACSDiva (v9.1; BD Biosciences), and data were analysed using FlowJo software (v10.8.1; BD Biosciences).

### In vivo studies for intravesical administration

There are a few mouse tumor models used to replicate the NMIBC disease features and assess the efficacy and combinability of experimental medicines. The most used is an orthotopic bladder cancer model where the MB49 bladder tumor cell line is instilled in the bladder. This model has been used by uro-oncologists for more than 30 years as it more closely mimics the features of the human disease, expresses markers of high grade NMIBC [17–19], and it has been extensively used to assess the preclinical efficacy of BCG therapy. This mouse tumor model is especially used to study the mechanism of resistance to BCG therapy and investigate the efficacy of novel treatments.

MBT2 mouse bladder cancer model is the second model used by uro-oncologists [20, 21]. The MB49 and MBT2 mouse tumor models are used interchangeably, however, MBT2 requires the use of C3H mice which have a predisposition for autoimmunity and allergy, therefore better suited for this type of investigation is the MB49 tumor model using C57BL6 mice.

Another available model in mice is the spontaneous generation of bladder cancer through exposure to a carcinogenic agent like hydroxybutylnitrosamine through drinking water. This model has been used to assess the activity of anktiva combination with BCG in addition to the MB49 model. However, the spontaneous cancer generation model carries major disadvantages: (1) the long experimental times, usually periods between 5 and 12 months (140 days for anktiva + BCG), (2) great heterogeneity across mice with some mice never developing a tumor, (3) treatments start usually before the tumor is detectable making it extremely challenging to link the lack of tumor growth to the treatment efficacy, and (4) it is representative of MIBC [22].

Due to the limitations of the MBT2 and the use of carcinogenic agent, the MB49 orthotopic bladder cancer model was chosen to assess the combinability of murinized PD1-IL2v with BCG.

### Murine orthotopic bladder tumor model

Female HuPD-1 transgenic C57BL/6 J mice (10–12 weeks old, weighing about 20 g, Charles River Laboratories, France) were anesthetized with ketamine/xylazine (80/15 mg/kg) and kept in dorsal position. Murine bioluminescent MB49-Luc cells (5*10^4^ cells in 100 µl of DPBS with Ca^2+^/Mg^2+^) were instilled in the murine bladder using a 24-gauge catheter (381212, BD Biosciences). Thirty minutes later the catheter was removed, and mice were returned to their cage.

The termination criteria were based on weight loss > 20%, calculated from mouse weight at the start of the first intravesical instillation, or animal well-being. All the procedures involving mice were approved (IACUC #1430).

### Assessing tumor volume by ultrasound imaging

Ultrasound imaging was acquired using the US system Vevo3100 (FUJIFILM VisualSonics, Inc.,Toronto, ON), and the linear US transducer array Mx550D that consists of 256 elements with a nominal center frequency of 40 MHz. During volumetric US acquisition, a stepper motor was used for the linear translation of the US transduce; the linear stepper motor moves in steps of 0.2 mm while capturing 2D parallel images, for a maximum 3D range distance of 6.4 cm. Tumor volume was estimated using the Vevo^®^Lab software (FUJIFILM VisualSonics, Inc.,Toronto, ON) [17, 18].

### Preparation and intravesical instillation of treatments

Eleven days after the MB49-Luc cells instillation, the tumor growth was monitored by ultrasound imaging and mice with tumor were randomly divided into the different treatment groups. Mice were treated once a week with either 100 µl of (i) BCG (OncoTICE, MSD, range of concentration 8–32 × 10^6^ CFU/ml) in 0.9% NaCl, or (ii) BCG with murinized PD1-IL2v (100 ng in 5% of glucose, P1AE2791, Roche), or (iii) BCG with murinized anktiva-like molecule (100 ng in 0.9% NaCl, P1AM2902).

To protect the operator and the working environment from contamination derived from BCG, Luer lock adapters were used: Microclave Clear Connector (cod 011-MC100, ICU Medical), Spiros Closed Male Luer (CH2000S-PC, ICU Medical) and 1 mL Luer-Lock Syringe (309628, BD Biosciences). For each treatment, animals were anesthetized with ketamine/xylazine (80/15 mg/kg) and kept in dorsal position; the catheter was inserted into the bladder, and the bladder was drained completely. The syringe containing 300 µl of treatment was connected to the catheter through the ‘safety system’ and the solution (100 µl/animal) was instilled into the bladder. The treatment was retained on the bladder for 1 h by keeping the catheter in place, then the catheter was removed and the animal returned to the cage under a warm red light to recover spontaneously from the anesthesia.

Mice were weighed daily and observed for the presence of pain signals, such as abnormal weight loss, ruffled hair coat and changes in the posture, and the tumor growth was evaluated twice a week by ultrasound imaging. Animals were sacrificed using a carbon dioxide chamber.

#### Treatments

In-vitro/ex-vivo: eciskafusp alfa, human PD-1 fused to a human IgG1 Fc portion contatining the P329G LALA mutation linked to a human IL-2v (huPD-1-huIgG1- P329G LALA-huIL-2v); anktiva-like molecule composed of a human dimeric IL-15 with N72D mutation and a human dimeric IL-15Rα sushi domain IgG1 Fc fusion protein,

In-vivo: murinized PD1-IL2v composed of the human PD-1 antibody fused to a murine IgG1 Fc portion containing the DAPG mutation, to prevent antibody-dependent cytotoxicity and antibody-dependent phagocytosis effector functions, linked to a murine IL-2v (huPD-1-muIgG1-DAPG-muIL-2v); murinized anktiva-like composed by a human dimeric IL-15 with N72D mutation and a human dimeric IL-15Rα sushi domain fused to the Fc portion of a murine IgG2a Fc fusion protein.

### BCG

Lyophilized BCG (Oncotice) was purchased from MSD Italia S.r.l. (Milan, IT), which after reconstitution as per instructions with sterile saline solution (0.9% NaCl) contains 0.4-1.6 × 10^7^ CFU/ml.

### Time to Positivity (TTP)

The MGIT (Mycobacteria Growth Indicator Tube) system was used to assess the bacterial load in the samples. Each MGIT tube contained 7 mL of 7H9 broth and an oxygen-sensitive fluorochrome embedded in silicone at the bottom. As bacteria grew, they consumed oxygen and produced carbon dioxide, leading to a reduction in oxygen levels. This depletion triggered the fluorochrome to fluoresce, with the fluorescence intensity increasing as oxygen was consumed. The increase in fluorescence was automatically detected by the BACTEC™ 960 instrument, which calculated the time to positivity (TTP) for each tube, reflecting the bacterial load in the samples. For each experiment, two MGIT tubes were enriched with 800 µL of OADC supplement and inoculated with 100 µL of the 10⁻² and 10⁻⁴ dilutions, prepared in DPBS. The MGIT tubes were then placed into the BACTEC™ 960 instrument until they became positive, and the TTP for each tube was recorded.

### Immuno-phenotyping of Tumor Infiltrating Lymphocytes (TILs) in the orthotopic murine model of NMIBC

At sacrifice tumor was analyzed for the immuno-phenotype of TILs. Tumor was digested in 3 ml of Digestion Buffer (Collagenase ABD 0,5 mg/ml, DNase I 100 mg/ml and Hyaluronidase 1 mg/ml; Collagenase A: cod. 10103578001 Merck Life Science, Collagenase B: cod. 11088807001 Merck Life Science, Collagenase D: cod. 11088858001 Merck Life Science, DNase I: cod. 11284932001 Merck Life Science, Hyaluronidase: cod. H3506 Merck Life Science), at 37 °C for one hour on a tube rotator, then filtered through a 70 μm cell strainer (cod. 352350, Corning) and smashed with 5 ml syringe piston. The filter was washed with 15 ml of RPMI + 10% FBS and digested samples were centrifuged at 1200 rpm for 10 min. The supernatant was discarded; the cell pellet was resuspended with FACS Buffer and counted with TC20 Automated Cell Counter (Bio-rad). The samples were then divided into appropriate tubes for FACS analysis. Two different protocols were conducted according to the marker analyzed (Supplementary Table S2):

I) To analyze cytokine production cells were first stimulated with Phorbol 12-myristate 13-acetate (0.1mM, PMA: cod. P8139 Merck Life Science) and Ionomycin (2 mg/ml, Ionomycin: cod. HY-13434, Med Chem Express) in the presence of BD GolgiStop™ Protein Transport Inhibitor (cod. 554724, Becton Dickinson) at 37 °C. Four hours later cells were stained with surface markers (CD45, TCRb, CD4, CD8, Tim-3 and viability dye), fixed, permeabilized and stained for intracellular markers (PD-1, IFNγ, TNFα and GrzB).
II) To identify Tregs, cells were first stained with surface markers (CD45, TCRb, CD4, CD8, and viability dye) and then processed using the eBioscience™ Foxp3 / Transcription Factor Staining kit (cod: 00-5523-00 Thermo Fisher Scientific) as per manufacturer instruction. Antibodies used for intracellular/intranuclear stain are PD-1, FoxP3 and TCF-1.

Gating strategy for Stain I and Stain II is reported in Supplementary Figure S1. Acquisition was carried out with Cytoflex LX (Becton Dickinson), samples were analyzed with FlowJo software (v10.8.1; BD Biosciences) and data and statistical analysis performed with GraphPad Prism 10.

### Statistical analysis

Log-rank test was used to compare survival distributions between two groups. To evaluate the robustness of treatment effects and account for potential confounding by initial tumor burden, baseline tumor volume (µL) was included as a continuous covariate together with treatment group in the model. Hazard ratios (HRs) with 95% confidence intervals (CIs) were estimated, and statistical significance was assessed using two-sided Wald tests. Continuous variables were expressed in box plot, showing min to max values, medians and interquartile ranges (Q1, Q3). To compare more than two groups based on one independent variable (i.e., tumor volume) the Kruskal-Wallis test with Dunn’s multiple comparison test was used. Dose–response curves were compared using a global F-test in nonlinear regression to assess differences between treatments and EC50 shifts. Statistical test was considered significant with *p* < 0.05.

## Results

### Eciskafusp elicits more potent IL-2R signaling and stronger effector functions than an anktiva-like molecule in in-vitro assays

**Fig. 1:**
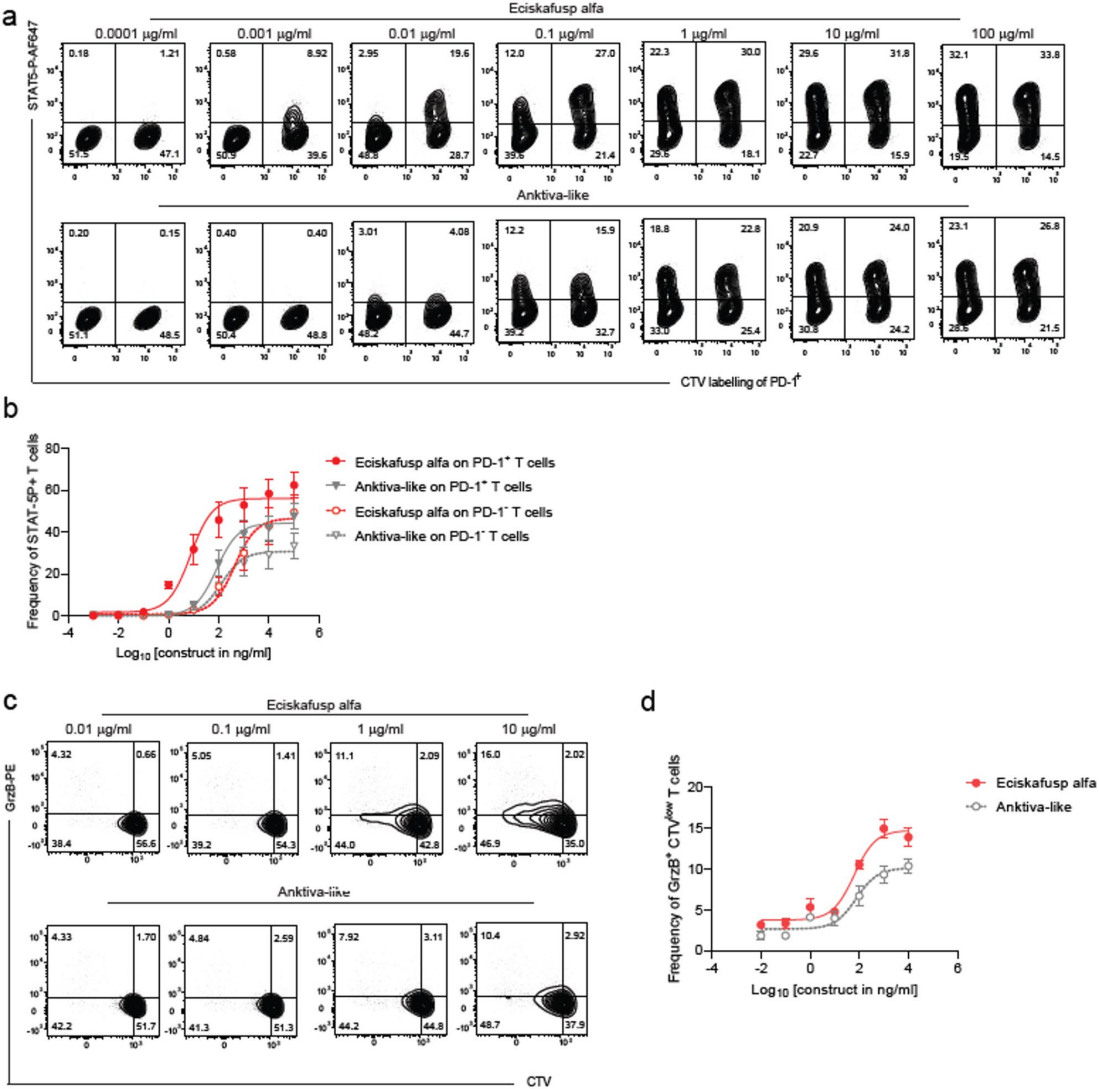
PD-1 mediated IL2R signaling and downstream cytotoxic effector function of eciskafusp alfa and anktiva-like molecule. (**A**) Representative FACS plot and (**B**) dose-response analysis (EC50 shift) of IL-2R signaling as STAT-5P of a coculture of PD-1^+^ and PD-1^−^ CD4^+^ T cells exposed for 15 min to increasing concentrations of either eciskafusp alfa or anktiva-like molecule (*p* = 0.0003 for PD1^+^ cells IL-2R signaling upon eciskafusp alfa vs. anktiva-like ; *p* < 0.0001 for IL-2R signaling upon eciskafusp alfa on PD-1^+^ and PD1^−^ cells). (**C**) Representative FACS plot and (**D**) dose-response analysis (EC50 shift) of cytotoxic effector functions of allogeneic CD4^+^ T cells cocultured for 5 days with human urothelial bladder cancer cell line 647-V in presence of increasing concentrations of either eciskafusp alfa or anktiva-like molecule (*p* = 0.015)

The relative potency of eciskafusp alfa and anktiva-like molecule was tested as their ability to signal through the IL-2R in cis on activated PD-1^+^, meaning on the same cell upon docking to PD-1, and on PD-1^−^ T cells cultured together, and therefore in trans. Eciskafusp alfa was 20 fold more potent in signaling through IL-2R on PD1^+^ T cells (in cis) than anktiva-like molecule (*p* = 0.0003; Fig. 1A and B; Table 1). In addition, eciskafusp alfa was 50-fold more potent in signaling on PD-1^+^ than on PD1– T cells (*p* < 0.0001) (Table 1), consistent with its cis-activity [23]. As expected, anktiva-like molecule did not show any cis-activity given it had the same activity on PD-1^+^ and PD-1^−^ T cells, regardless of PD-1 expression, and was roughly 2-fold more potent on PD-1^−^ T cells than eciskafusp alfa due to the N72D mutation, which increases the affinity of IL-15 for the beta subunit of the IL-2 receptor (Fig. 1A and B; Table 1).

**Table 1:** EC_50_ and fold increase/decrease of IL-2R signaling (STAT-5P+) in presence of increasing concentrations of either Eciskafusp Alfa or an anktiva-like molecule

Molecule	EC_50_ PD-1^+^	EC_50_ PD-1^−^	Potency reduction on PD-1^+^ relative to eciskafusp alfa	Potency reduction on PD-1^−^ relative to anktiva	Cis-activity window
**Eciskafusp alfa**	46.64	2635	1	1.9	56. 57
**Anktiva-like**	903	1390	19.36	1	1.54

To study the effect of eciskafusp alfa on T cell effector functions against human urothelial bladder cancer, CTV labeled CD4^+^ T cells from healthy donors were cocultured for 5 days in presence of the 647-V human cell line with increasing concentrations of either eciskafusp alfa or anktiva-like molecule. Allo-reactive (CTV^low^) CD4^+^ T cells were assessed for their expression of Granzyme B (GrzB), used as a marker for cytotoxic effector functions. Eciskafusp alfa induced a significantly stronger CD4⁺ T cell effector response (*p* = 0.015), as demonstrated by the greater area under the curve, compared to anktiva-like molecule (Fig. 1C and D; Table 2). The greater activity of eciskafusp alfa was attributed to the PD-1–mediated cis-delivery of IL-2R signaling to alloantigen-specific PD-1⁺ CD4⁺ T cells.

**Table 2:** EC_50_ and AUC of proliferating and granzyme B secreting CD4 T cells towards the human urothelial bladder cancer cell line 647-V

	EC_50_	Total area
**Eciskafusp alfa**	5620	76,377
**Anktiva-like**	5857	53,862

Data are from 5 days coculture in the presence of increasing concentrations of either eciskafusp alfa or an anktiva-like molecule

These observations indicate a functional differentiation between eciskafusp alfa and anktiva-like molecules, or in general untargeted IL-2Rβγ biased cytokines, due to the PD-1 mediated cis-delivery of IL-2R agonism of eciskafusp alfa leading to the preferential targeting, expansion and effector function of PD-1^+^ antigen-specific T cells [23].

### Murinized PD1-IL2v boosts the efficacy of BCG in orthotopic non-muscle invasive bladder cancer

**Fig. 2:**
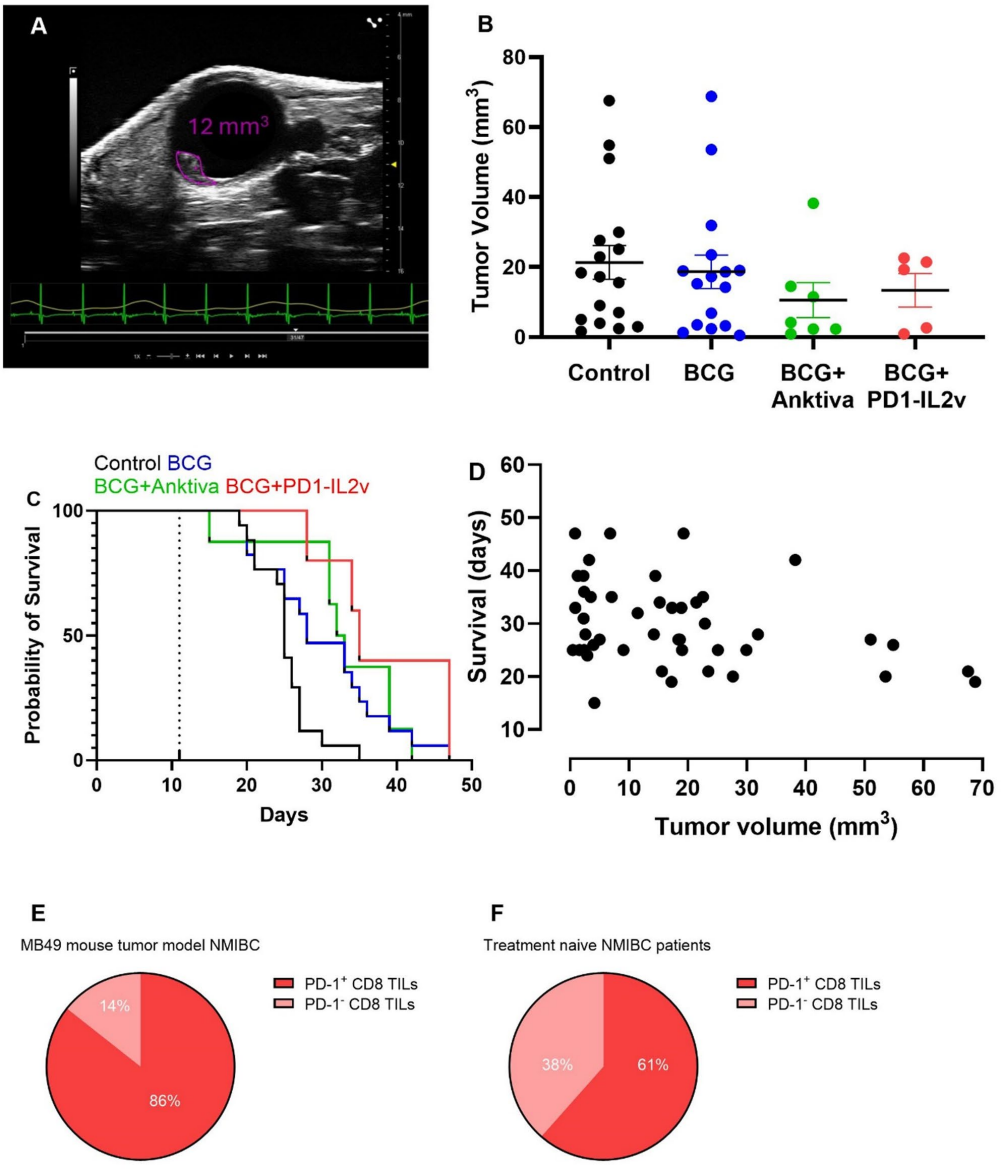
Survival curves from in-vivo efficacy study using MB49 mouse model of NMIBC in hu-PD-1 mice. (**A**) Axial frame of the murine bladder with a tumor detected by ultrasound imaging 11 days after the intravesical instillation of the MB49 cells, with a representative image from an animal with tumor volume of 12 mm^3^ highlighted in purple. (**B**) Tumor volume at the time of the intravesical instillation of treatment, day 11 (bars show mean and SEM, each dot indicates a single mouse). (**C**) Survival analysis shown as Kaplan-Meier curve. The dotted line shows the first treatment (day 11), followed by 3 additional weekly treatments. The termination criteria were based on weight loss > 20%, calculated from mouse weight at the start of the first intravesical instillation, or animal well-being. (**D**) Scatter plot showing baseline tumor volume versus survival time for individual mice. Each point represents one animal. (**E**) Frequencies of PD-1^+^ and PD-1^−^ within CD8^+^ TILs in MB49-derived murine orthotopic NMIBC (*n* = 5) and (**F**) from six treatment-naive patients with urothelial bladder cancer

The efficacy and combinability of murinized PD1-IL2v surrogate with BCG were assessed in the MB49 orthotopic bladder cancer model, a mouse tumor model for NMIBC responsive to anti-PD-1 therapy [24]. HuPD-1 transgenic mice were intravesically inoculated with MB49 cells, and once tumors became detectable (Fig. 2A) the mice were randomly assigned to various treatment groups. The randomization procedure used did not constrain group sizes, and the study design included two larger control groups (i.e., untreated and BCG treated) to provide a robust baseline for comparison. The treated groups had fewer animals because the focus was on detecting treatment effects, not estimating baseline variability, and kept smaller in compliance with animal ethics guidelines. The distribution reflects the planned design to detect biologically relevant effects rather than a flaw in the randomization process, with pre-treatment tumor size similar among groups (*p* = 0.46) (Fig. 2B). In this in-vivo experimental setup, we used a murinized PD1-IL2v composed of the human PD-1 antibody fused to a murine IgG1 Fc portion containing the DAPG mutation, to prevent antibody-dependent cytotoxicity and antibody-dependent phagocytosis effector functions, linked to a murine IL-2v (huPD-1-muIgG1-DAPG-muIL-2v). Mice were treated once a week for 4 weeks with intravesical BCG alone or in combination with murinized PD1-IL2v. For benchmarking purposes, a murinized anktiva-like molecule, constituted by a human IL-15Ra sushi and a human IL-15 containing the N72D mutation fused to the Fc portion of a murine IgG2a (hu IL15Ra sushi – hu IL15 N72D – muIgG2a wt Fc), was also used in combination with BCG.

Analysis of the survival curves demonstrated the therapeutic efficacy of BCG monotherapy (*p* = 0.037 vs. control), and of the combination therapies BCG with murinized anktiva-like molecule (*p* = 0.0062 vs. control) or BCG with murinized PD1-IL2v (*p* = 0.0065 vs. control) (Fig. 2C). The analysis of the two combination therapies, BCG with murinized anktiva-like molecule and BCG with murinized PD1-IL2v, demonstrated comparable efficacy in terms of animal survival to 33 and 35 days, respectively (Fig. 2C, p = n.s.). Both combination therapies did not show improved animal survival vs. BCG alone (Fig. 2C, p = n.s.), as previously reported for BCG in combination with anktiva [25]. In a Cox proportional hazards model including baseline tumor volume as a covariate, treatment remained significantly associated with improved survival (HR = 0.29, 95% CI 0.14–0.61, *p* = 0.0012), while baseline tumor volume showed no independent significant association with survival (HR = 1.02, 95% CI 1.00-1.04, *p* = 0.081) (Fig. 2D).

Because the in-vitro studies (Fig. 1A-D) showed increased activity of eciskafusp alfa on PD-1^+^ T cells respect to anktiva-like molecule, we compared the frequencies of PD-1^+^ CD8^+^ TILs in the bladder of NMIBC model versus the human disease. The majority of CD8^+^ T cells infiltrating the murine tumor expressed PD-1 (86%) (Fig. 2E), as opposed to the human disease setting where 61% of the CD8^+^ TILs were PD-1^+^ (Fig. 2F). Therefore, in this mouse tumor model both the murinized PD1-IL2v and murinized anktiva-like molecule will target the same PD-1^+^ CD8^+^ TILs population regardless of the cis- versus trans-activity, making it challenging to report a difference in the efficacy of the two molecules. In addition, we demonstrated that murinized PD1-IL2v did not affect BCG viability when administered in combination (Supplementary Figure S2), as reported for anktiva [26].

### Functional phenotype of CD8^+^ T cells in the tumor mass upon treatment

**Fig. 3:**
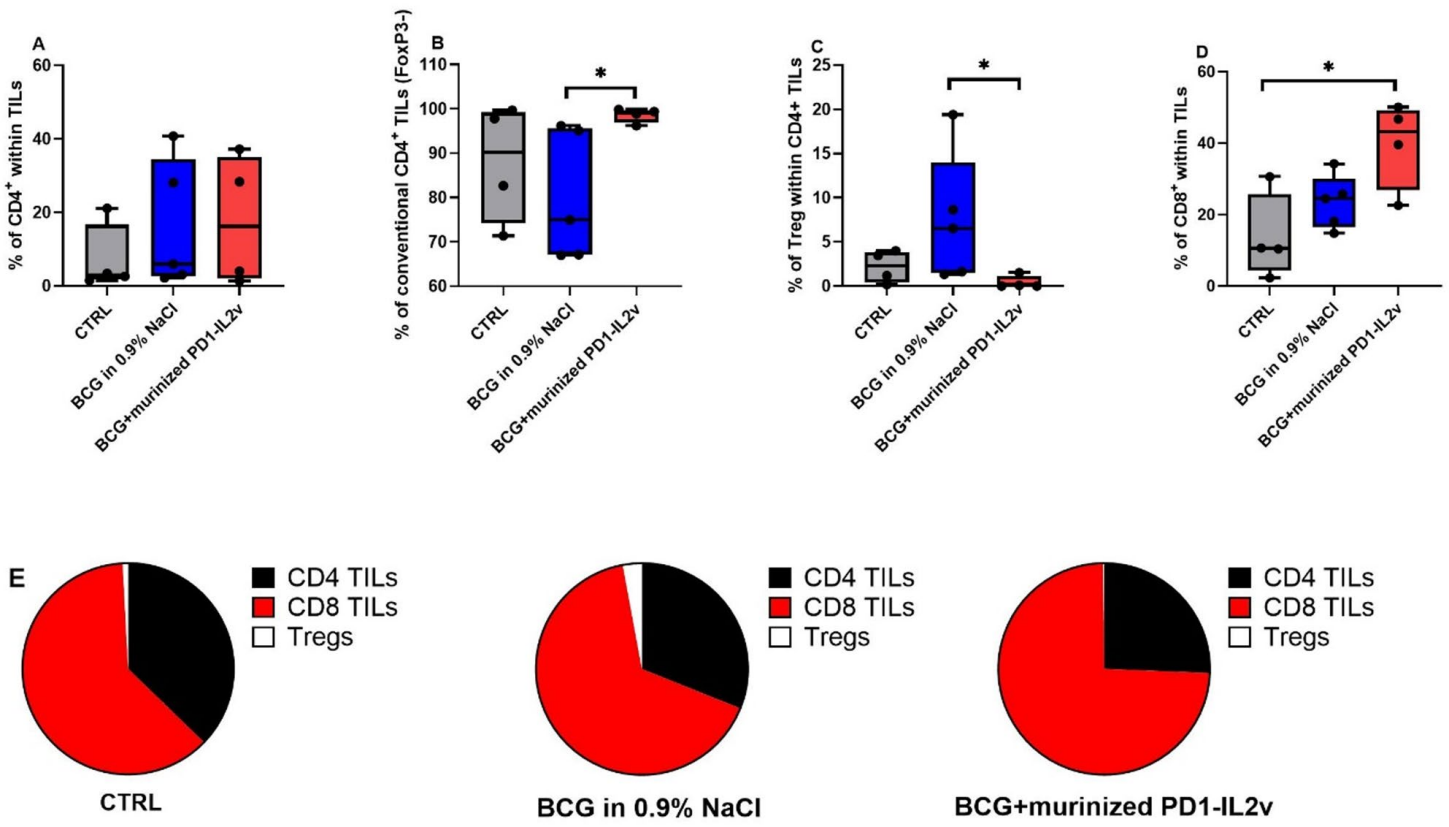
CD8^+^ T-cell expansion in murine orthotopic bladder cancer after combined BCG and murinized PD1-IL2v therapy. Frequency of CD4^+^ T cells (**A**), conventional (FoxP3-) CD4^+^ T cells (**B**), Tregs (**C**) and CD8^+^ T cells (**D**); frequency was calculated on live CD45^+^CD3^+^ cells from disaggregated tumor. Each dot indicates a single mouse. *p*-value: * = <0.05. The frequency of TIL sub-populations was averaged across animals, and the means were visualized in the pie chart to show how each subpopulation contributes proportionally to the entire TIL population (**E**)

To investigate whether the therapeutic effects of BCG alone and in combination with murinized PD1-IL2v were mediated through distinct immune cell types, we conducted immuno-pharmacodynamic analyses on TILs isolated from orthotopic tumors of control animals, those treated with BCG only and BCG in combination with murinized PD1-IL2v. Tumor pseudo-progression (i.e., enlargement of tumor lesions) has been described in patients receiving cancer immunotherapies, such as immune checkpoint inhibitors and cytokine therapies, where treatment-induced immune cell infiltration and inflammation transiently increase tumor size without representing true disease progression [27]. This phenomenon would make normalization of immune cell sub-populations to tumor size poorly reliable. Thus, to capture treatment-induced changes in immune composition the immune subsets were normalized to CD45^+^CD3^+^ cells. We found that the treatments with BCG, and the combination of BCG with murinized PD1-IL2v did not differently modulate the overall frequency of total CD4^+^ T cells in TILs (Fig. 3A). However, the combination of BCG with murinized PD1-IL2v significantly increased the percentage of conventional (FoxP3^-^) CD4^+^ T cells when compared to BCG alone (Fig. 3B, mean ± SEM, 98.6 ± 0.8% vs. 80.1 ± 6.5%, *p* = 0.0385). The proportion of Tregs within the tumor was elevated in BCG-treated animals compared to controls (mean ± SEM, 7.5 ± 3.2% vs. 2.2 ± 0.9%, *p* = 0.023). Notably, Treg frequency in the BCG plus murinized PD1-IL2v group was markedly reduced relative to BCG monotherapy (mean ± SEM, 0.4 ± 0.4% vs. 7.5 ± 3.2%, *p* = 0.0193) (Fig. 3C). These findings suggest that, within the CD4⁺ lymphocyte immune compartment, murinized PD1-IL2v in combination with BCG preferentially promotes the expansion of conventional CD4⁺ lymphocytes over Tregs, consistent with the target selectivity of eciskafusp alfa reported previously for CD8^+^ TILs [23].

In addition, we observed a significant expansion of the frequency of CD8^+^ TILs for the combination of BCG with murinized PD1-IL2v versus control (mean ± SEM, 40 ± 6% vs. 13 ± 6%, *p* = 0.0139), but not in BCG monotherapy (mean ± SEM, 23 ± 3%, *p* = 0.372 vs. control) (Fig. 3D). Given that in this murine model most CD8^+^ TILs express PD-1 (Fig. 2D), the expansion observed upon each treatment involved CD8⁺ PD1⁺ cells (Supplementary Figure S3).

To better capture the impact of the therapies on TIL populations, the relative frequencies of the three subsets are reported in a pie chart. We found a reduction in Tregs in favor of an expansion of CD8^+^ T cells in the group treated with BCG in combination with murinized PD-IL2v versus BCG monotherapy and the control group (Fig. 3E). Overall, this finding confirms the preferential targeting of CD8^+^ TILs over Tregs by eciskafusp alfa, as previously described for its systemic administration [23]. Conversely, BCG monotherapy increased both CD8^+^ and Treg counts within TILs supporting the notion of leading an unspecific stimulation, as reported in the clinical setting [28]. This unspecific stimulation may limit the overall efficacy of BCG since it relies on the baseline levels of infiltrating CD8^+^ TILs over Tregs in the patient primary tumor, potentially explaining why 50% of the patients do not benefit from the treatment [28].

**Fig. 4:**
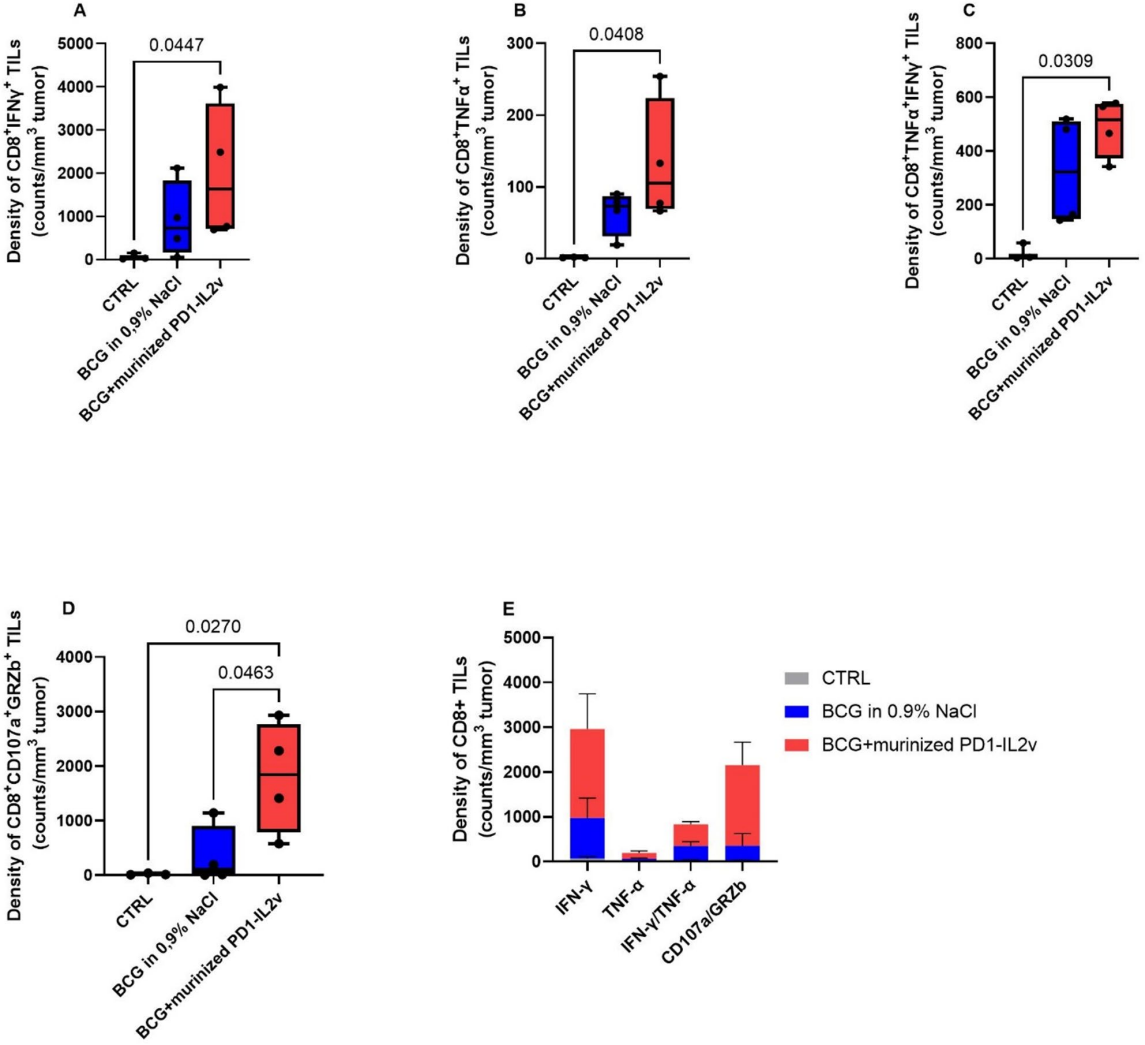
BCG in combination with murinized PD1-IL2v expands polyfunctional effector CD8^+^ TILs. Density of CD8^+^ TILs expressing the cytokines IFN-γ (**A**), TNF-α (**Β**), IFN-γ/ TNF-α (**C**), and positive for the degranulation marker CD107a and expressing Granzyme B indicating an active degranulation (**D**). Each dot represents a single mouse. Grouped column chart that compares density of polyfunctional CD8^+^ TILs expressing cytokines and GrzB across different experimental groups (E), with each group consisting of all animals reported in panels **A**-**D** (bars show the mean ± SEM)

Compared with untreated tumors, a deeper phenotypical and functional analysis of the TILs revealed that only the combination of murinized PD1-IL2v with BCG markedly expanded the density of CD8^+^ TILs able to secrete IFNγ, TNFα or both, together with Granzyme B (Fig. 4A and D), without modifying the expression level of the cytokines or Granzyme B (Supplementary Figure S4). These findings suggest that the combination of BCG with murinized PD1-IL2v drives the expansion of polyfunctional CD8^+^ TILs rather than leading to an increased functionality of the target cells upon local co-administration (Fig. 4E).

### Human bladder CD8 ^+^ TILs express higher amounts of PD-1 receptors per cell compared to CD8⁺ T cells derived from peripheral blood

The key requirements for eciskafusp alfa to have a differentiated mechanism of action from trans-acting fibroblast activated protein (FAP)-IL2v and other untargeted or tumor/stroma targeted IL-2Rβγ-biased cytokines are (1) the presence of CD8^+^ TILs in the tumor and (2) the expression of PD-1 on their surface.

**Fig. 5:**
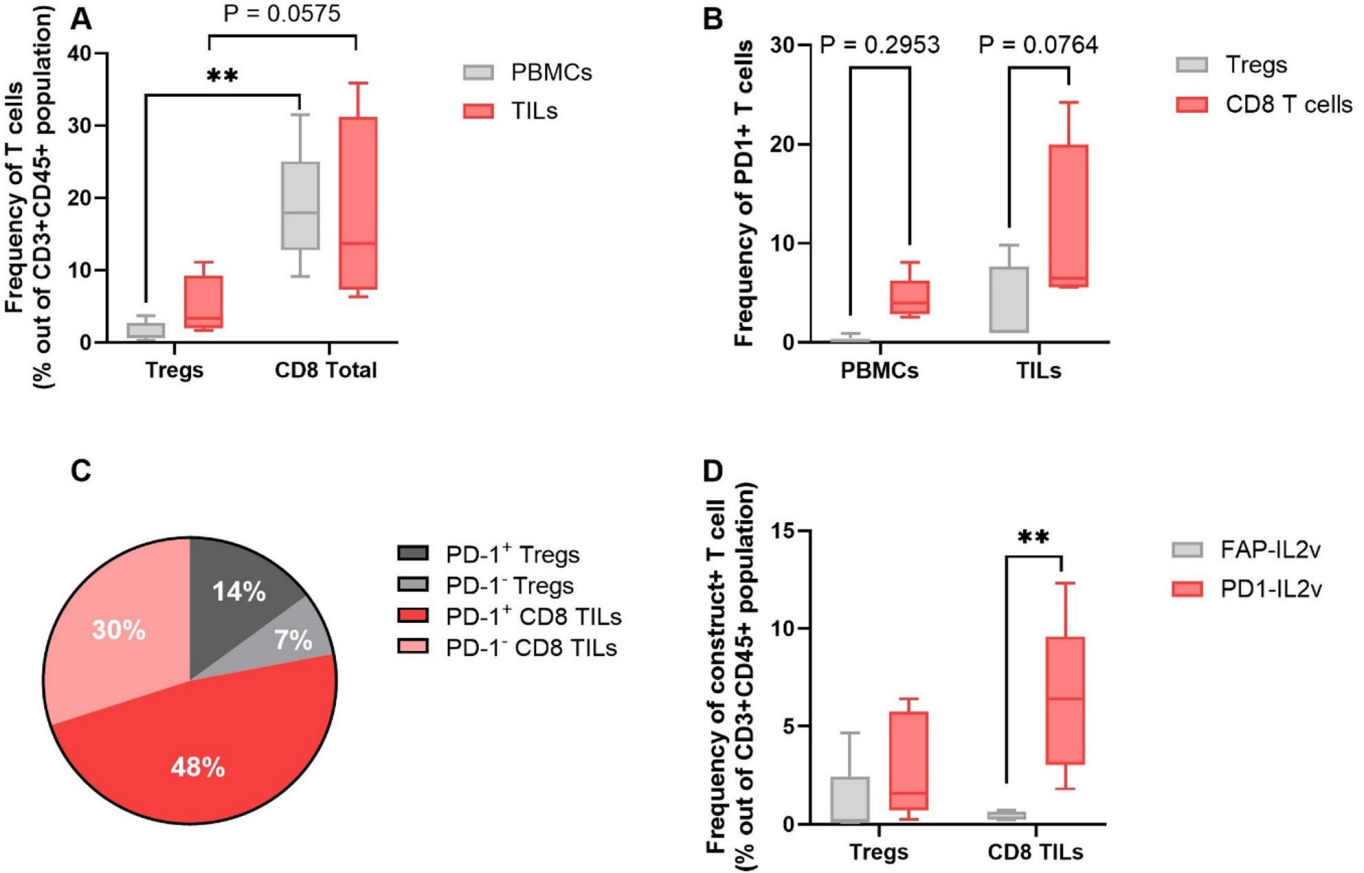
Frequencies of CD8 + T cells and Tregs showing eciskafusp alfa preferential binding to CD8 + TILs. (**A**) Frequency of CD8^+^ TILs and Tregs among CD45 + CD3+ cells in the tumor and blood of six bladder cancer patients. (**B**) Frequency of PD1 + Tregs and CD8 + T cells among CD45 + CD3+ cells in PBMC and TILs of six bladder cancer patients. (**C**) Frequency of PD-1^+^ and PD-1^−^ T cells within CD8^+^ TILs and Tregs in six bladder tumors. (**D**) Binding behavior of eciskafusp alfa and FAP-IL2v to CD8^+^ TILs versus Tregs

Previously, with the purpose of better understanding the specific tumor tropism of murinized PD1-IL2v, we isolated leukocytes from the blood and tumors of huPD-1 transgenic mice bearing Panc02 tumors and measured ex-vivo the frequencies and amounts of PD-1 and IL-2Rβ on T cells [23]. We found that, although the frequencies of T cell subsets expressing PD-1 on their surface were quite similar in both blood and tumors, in the tumors an effector memory population of CD8^+^ T cells was found to express roughly 15.000 PD-1 receptors per cell. Interestingly, in the blood, the same population expressed roughly 700 PD-1 receptors per T cell, like other T cell subsets, including CD4 ^+^ T_EM_ and T_regs_. On the other hand, the IL-2Rβ frequencies and the receptor number per T cells were similar on CD8^+^ T cells in the tumor and blood [23]. This different expression profile of PD-1 and IL-2R on T cells in the blood and tumor supports the observed differences in targeting and activity between murinized PD1-IL2v and an untargeted or tumor/stroma targeted IL-2Rβγ-biased cytokines [23]. To explore if eciskafusp alfa could find application to the human settings, single cell suspensions of tumor biopsies or tumor resections of treatment-naive patients with bladder cancer have been assessed for the frequencies of CD8^+^ TILs and the expression levels of PD-1 in addition to the ex-vivo binding behavior of eciskafusp alfa. We found detectable frequencies of CD8^+^ TILs (*n* = 6, mean ± SEM= 17.4 ± 6.5%), higher than the frequencies of Tregs (*n* = 6, mean ± SEM = 4.87 ± 2.12%), among hematopoietic cells in patient derived tumor samples (Fig. 5A). Furthermore, 10 ± 4.52% of the total CD8^+^ TILs and 3.25 ± 2.18% of the Tregs were PD-1^+^ (Fig. 5B), corresponding to 61% of CD8⁺ TILs and 67% of Tregs (Fig. 5C). This finding was further corroborated by the observation on the preferential binding of eciskafusp alfa to CD8^+^ TILs over Tregs in comparison to FAP-IL2v (Fig. 5D). Altogether these data support the rationale for exploring the intravesical administration of eciskafusp alfa together with BCG in the treatment of patients with non-muscle invasive bladder cancer.

## Discussion

This study shows that in a preclinical mouse model of NMIBC, the intravesical instillation of BCG combined with murinized PD1-IL2v increases the survival of treated mice by boosting the expansion of CD8^+^ TILs with effector functions, without increase in the Treg subset as observed for BCG monotherapy. Thereof, the latter finding implies that the resistance to clinical BCG treatment might be caused by the local expansion of Tregs at detriment of CD8^+^ TILs, leading to potential treatment failure and long-term disease relapse. Conversely, we observed that intravesical combination of BCG with murinized PD1-IL2v prevents Tregs expansion while increasing the presence of polyfunctional cytotoxic CD8^+^ TILs, and these changes led to control of tumor growth based on an immune phenotype different from that found upon other therapeutic strategies.

To differentiate from an untargeted or tumor/stroma targeted IL-2Rβγ biased cytokine that acts in trans, eciskafusp alfa requires the presence of PD-1^+^ tumor specific CD8^+^ T cells in the tumor to act in cis (binding and signaling on the same cell). Herein, we demonstrate that human bladder cancer tumors are enriched for CD8^+^ T cells over Tregs and that 60% of these CD8 cells express PD-1. When we performed an ex-vivo binding competition on the same patient tumor samples, we observed a preferential binding of eciskafusp alfa to CD8^+^ TILs over Tregs as opposed to FAP-IL2v which bound to both populations indistinctively. In addition, in in-vitro studies, eciskafusp alfa was more potent on PD-1^+^ than on PD-1^−^ T cells in line with its cis-activity. As expected, the anktiva-like molecule did not show any cis-activity as it was equipotent on PD-1^+^ and PD-1^−^ T cells.

In an in-vivo preclinical model of NMIBC, the BCG monotherapy and its combination with murinized anktiva-like molecule and murinized PD1-IL2v demonstrated comparable efficacy in increasing the survival of mice. However, the majority of tumor infiltrating CD8^+^ T cells expressed PD-1. Therefore, in this specific setting both the murinized PD1-IL2v and murinized anktiva-like molecule will target a similar population of CD8^+^ TILs with reduced influence of the cis- versus trans-targeting. In contrast, the cis-activity of eciskafusp alfa may provide greater benefit than anktiva in the clinical setting, where 61% of CD8⁺ tumor-infiltrating lymphocytes (TILs) express PD-1.

Despite the model limitations, immuno-pharmacodynamic studies of the TILs isolated from orthotopic NMIBC tumors revealed that murinized PD-IL2v in combination with BCG increased the frequency and amounts of CD8^+^ T cells and conventional CD4^+^ T cells, at the detriment of Tregs. Overall, this finding confirms the preferential targeting of CD8^+^ TILs over Tregs by murinized PD1-IL2v, as previously described for the systemic route of administration [23]. Conversely, BCG monotherapy increased both CD8 and Treg counts within TILs supporting the notion of leading an unspecific stimulation, potentially limiting the overall efficacy of BCG. Additionally, murinized PD1-IL2v in combination with BCG elicited the expansion of polyfunctional CD8^+^ TILs. This functional profile of CD8^+^ TILs shares similarities with the one found in intra-tumoral CD8^+^ T cells of mouse models treated systemically with murinized PD1-IL2v and associated with an improved efficacy [13, 23].

Overall, these data are indicative that while BCG treatment, by expanding Tregs in addition to CD8^+^ TILs, might promote long-term an adaptive resistance mechanism leading to disease relapse [29], its combination with murinized PD1-IL2v significantly increases the number of polyfunctional cytotoxic CD8^+^ TILs, a phenotype that is generally associated with improved antitumor activity [30, 31].

## Conclusions

The current findings suggest that clinical co-administration of intravesical BCG and eciskafusp alfa may help address some of the limitations of BCG monotherapy and could offer advantages over BCG combined with anktiva, which predominantly induces non-specific activation of NK cells and bystander CD8⁺ TILs. In contrast, eciskafusp alfa specifically targets and activates PD-1⁺ tumor-specific CD8^+^ TILs, while sparing Tregs. In line with these observations is the potential predictivity of the ratio CD8^+^ TILs/Treg for therapeutic response in neoadjuvant chemotherapy in muscle-invasive bladder cancer (MIBC) [32] and in breast cancer studies [33, 34].

These findings provide the rationale for exploring the clinical use of eciskafusp alfa for intravesical administration in high-risk NMIBC, with the combination of BCG and eciskafusp alfa potentially deployable for patients with low CD8^+^ TIL/Treg ratio at TURBT or as a second line of treatment for NMIBC patients unresponsive to BCG. Moreover, this approach may also allow for a reduction in the required dose of BCG, an important therapeutic consideration in times of BCG shortages.

## Supplementary Information

Supplementary Material 1.

## Data Availability

Data were generated by the authors and included in the article. The data generated in this study are available upon request from the corresponding author.
