# Supplementary material for: PD1-IL2v expands and induces effector CD8+ TILs, but not Tregs, in the BCG treated orthotopic non-muscle invasive bladder cancer model
Source: J Exp Clin Cancer Res. 2026 Feb 11;45:82. doi: 10.1186/s13046-026-03667-w (PMC13036890; doi:10.1186/s13046-026-03667-w)
Supplement: Supplementary file 1 — Supplementary Material 1. [file 13046_2026_3667_MOESM1_ESM.docx]

#### **PD1-IL2v boosts effector CD8+ TILs in the BCG treated orthotopic non-muscle invasive bladder cancer model**

Irene Locatelli, Marco Lorenzoni, Chiara Venegoni, Alessia Di Coste, Rita Sorrentino, Jithin Jose, Matteo Bellone, Daniela Cirillo, Patrick Weber, Amrita Manchala, Ralf J Hosse, Valeria Nicolini, Pablo Umana, Christian Klein, Andrea Salonia, Francesco Montorsi, Marco Moschini, Laura Codarri Deak, Massimo Alfano.

- Supplementary Table S1. Antibodies used for the staining of human PBMC.
- **Supplementary Table S2. Antibodies used for the staining of murine cells.**
- **Supplementary Figure S1. Mouse gating strategy.**
- **Supplementary Figure S2. Mycobacteria load in BCG preparations.**
- **Supplementary Figure S3. Expansion of CD8+PD1+ TILs.**
- **Supplementary File S4. Level of expression of IFNγ, TNFα and GRZb in CD8+ TILs.**

Supplementary Table S1. Antibodies used for the staining of human PBMC.

| **Marker** | **Fluorophore** | **Dilution** | **Clone** | **Company** | **Catalog No** |
| --- | --- | --- | --- | --- | --- |
| **Surface staining:** |  |  |  |  |  |
| Live Dead | APC-Cy7 | 1:1000 |  | ThermoFisher | 65-0865-18 |
| CD45 | AF700 | 1:100 | HI30 | BioLegend | 56-9459-42 |
| CD3 | BV605 | 1:100 | OKT3 | BioLegend | 317322 |
| CD4 | BUV496 | 1:100 | OKT4 | BioLegend | 612936 |
| CD8 | BUV395 | 1:100 | RPA-T8 | BD | 563795 |
| CD366 (TIM3) | BV711 | 1:20 | F38-2E2 | BioLegend | 345024 |
| CD218a | PerCPCy5.5 | 1:100 | 3DS223H | eBioscience | 313810 |
| CD56 | BV786 | 1:20 | 741182 | BD | 362550 |
| PD-1 | PE | 5 ug/ml | EH12.2H7 | Biolegend | 329906 |
| **After fixation:** |  |  |  |  |  |
| TCF1 | AF647 | 1:100 | C63D9 | CST | 6709S |
| PD-1 IC |  | 1:100 | D4W2J | CST | 86163S |
| anti-Rabbit secondary | BV421 | 1:100 | Polyclonal | BD Biosciences | 565014 |
| FOXP3 | PE-CF594 | 1:50 | 206D | BioLegend | 320126 |
| AF =Alexa Fluor; APC = allophycocyanin; BUV = brilliant ultraviolet; BV = brilliant violet; Cy = cyanine; FOXP3 = Forkhead box P3; PD‑1 = programmed cell death 1; PE = phycoerythrin; PerCP = peridinin-chlorophyll-protein; TCF = transcription factor; TIM-3 = T-cell immunoglobulin and mucin-domain containing-3. | | | | | |

**Supplementary Table S2. Antibodies used for the staining of murine cells.**

| **Protein** | **Fluorochrome** | **Clone** | **Catalogue** | **Provider** |
| --- | --- | --- | --- | --- |
| Live/Dead | Viakrome IR808 | - | C36628 | Beckman Coulter |
| CD45 | BUV395 | 30-F11 | 565967 | BD Biosciences |
| TCRb | APC-Cy7 | H57-597 | 109220 | Biolegend |
| CD4 | BUV496 | RM4-5 | 569180 | BD Biosciences |
| CD8 | BV510 | 53-6.7 | 100752 | Biolegend |
| TIM3 | AF647 | B8.2C12 | 134006 | Biolegend |
| CD107a | PE | 1D4B | 558661 | BD Biosciences |
| CD25 | PE-Cy7 | PC61 | 552880 | BD Biosciences |
| PD-1 | AF488 | 1D7D5W | 34920 | Cell Signaling |
| IFNg | AF700 | XMG1.2 | 557998 | BD Biosciences |
| TNFa | PE-Cy7 | MP6-XT22 | 506306 | Biolegend |
| GRZ b | BV421 | QA18A28 | 396414 | Biolegend |
| FOXP3 | BV421 | MF-14 | 126419 | Biolegend |
| TCF1/7 | PE | S33-966 | 564217 | BD Biosciences |

**Supplementary Figure S1. Mouse gating strategy.** Representative flow cytometry strategy for intratumoral T lymphocytes. Stain I cytokine shows gating strategy to identify PD1^+^, GRZb^+^CD107a^+^, IFNγ, TNFα and IFNγ^+^TNFα^+^ CD8 TILs. Stain II Tregs shows gating strategy for the identification of intratumoral Tregs identified as CD4^+^ T cells that are FoxP3^+^CD25^+^ (highlighted in the corresponding plot).

**
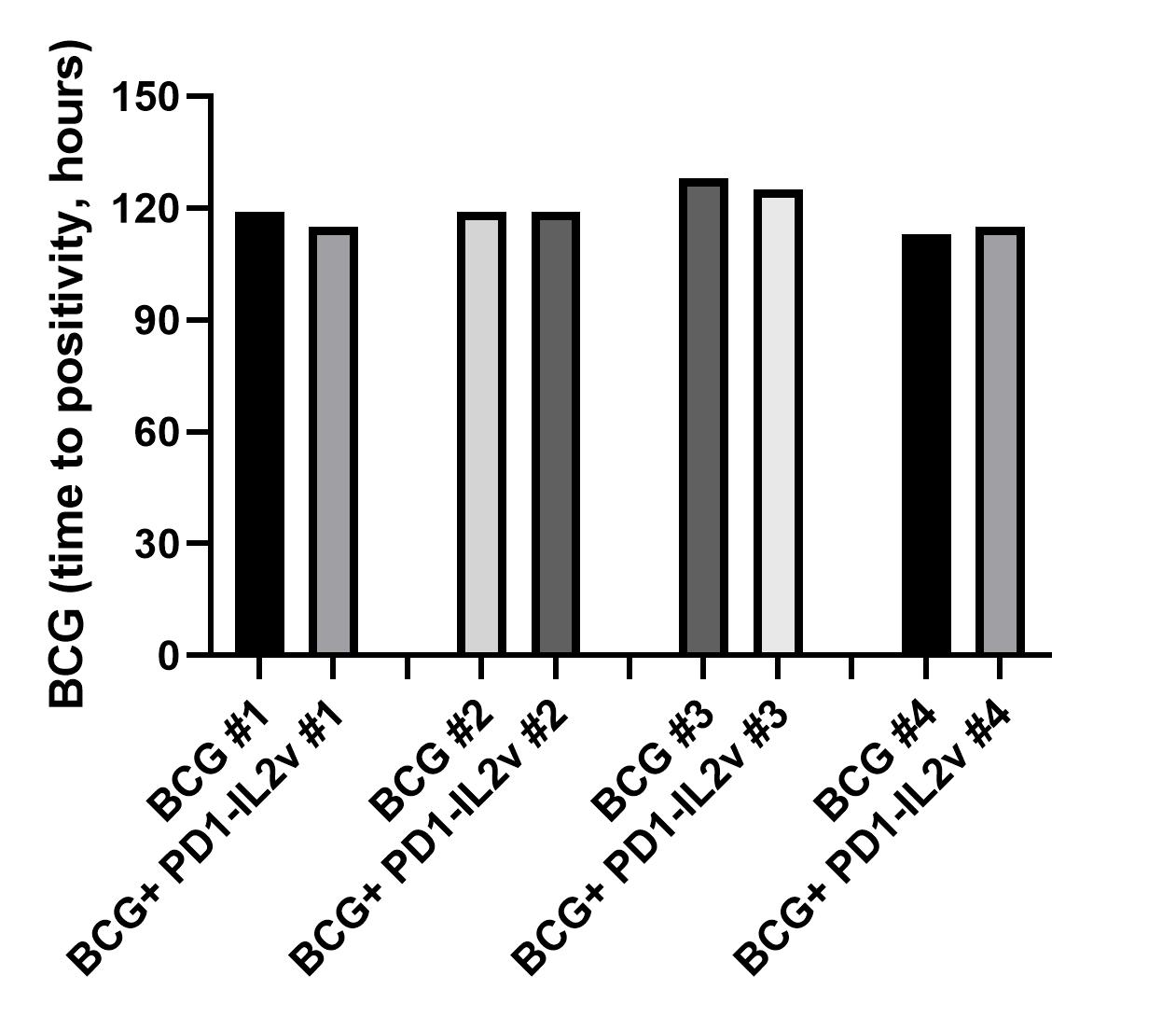
**

**Supplementary Figure S2. Mycobacteria load in BCG preparations.** The four preparations used for the four weekly intravesical instillations were assessed for the BCG time to positivity. The leftover of the preparations was evaluated with the MGIT system, as described in the methods section.


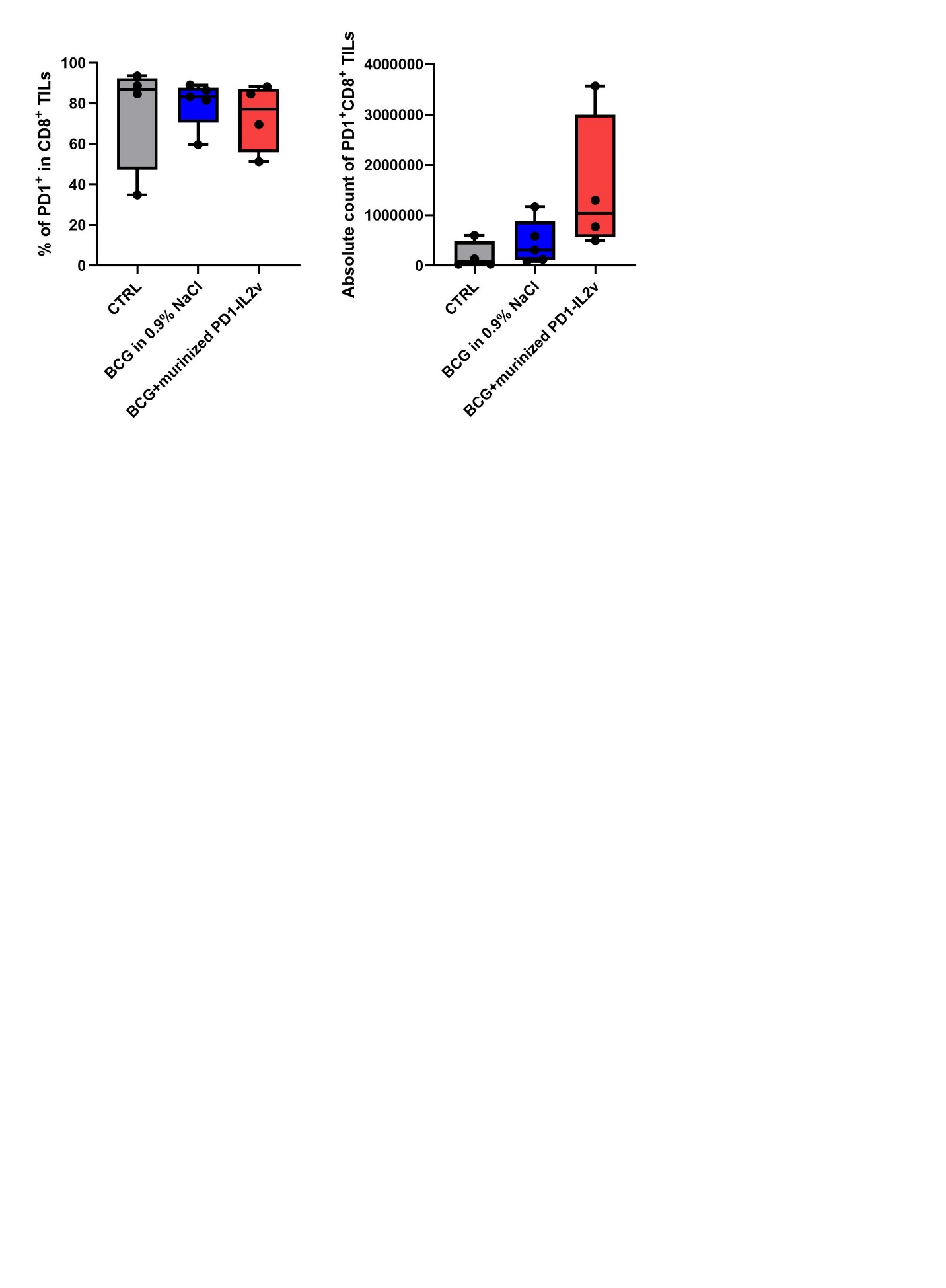


**Supplementary Figure S3. Expansion of CD8^+^PD1^+^ TILs.** Frequency and absolute count of CD8^+^PD1^+^ T cells; frequency was calculated on live CD45^+^CD3^+^ cells from disaggregated tumor, the absolute count was estimated using the frequency of the immune population multiplied by the total number of live cells counted after disaggregation of the tumor mass. Each dot indicates a single mouse.


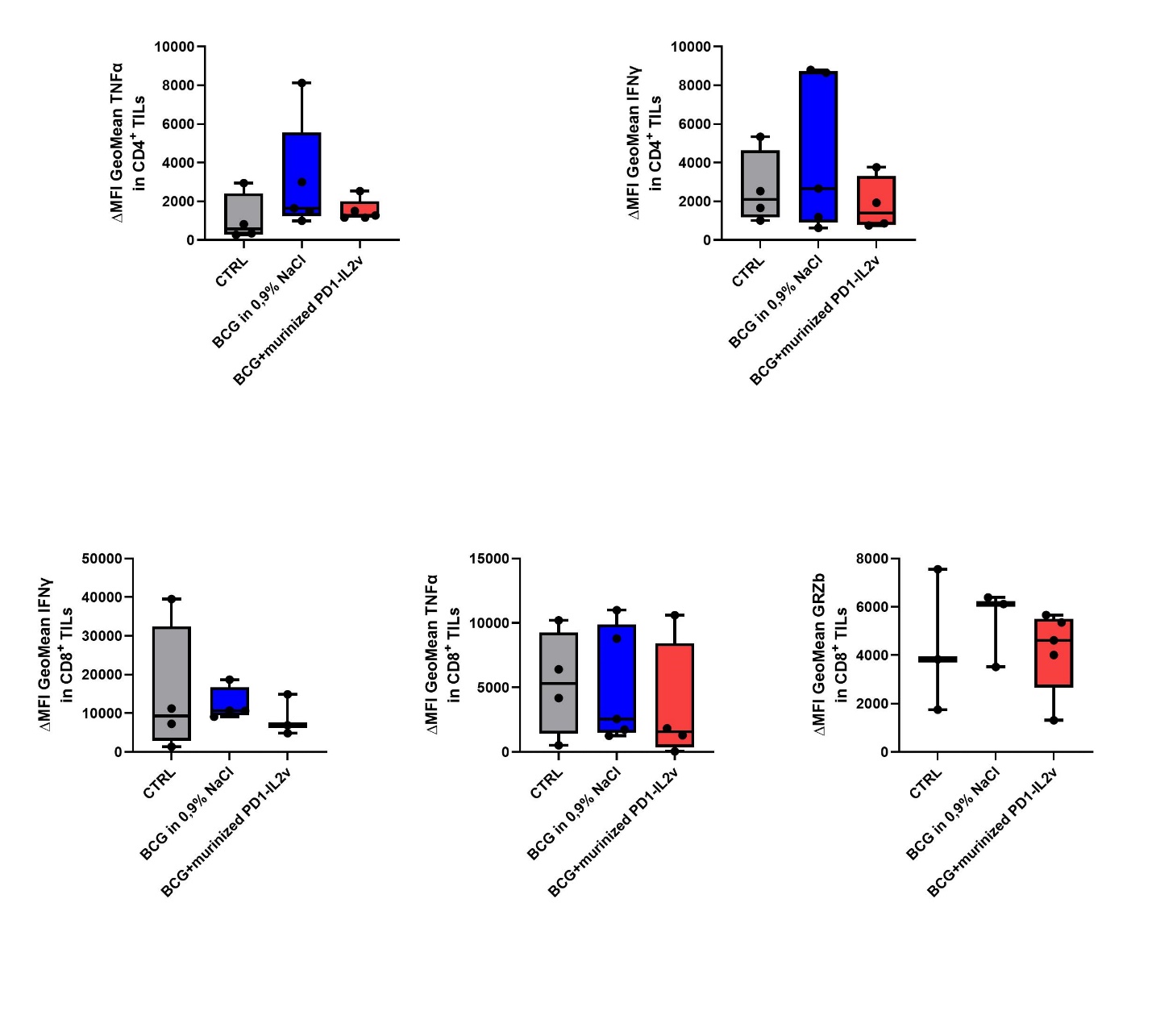


**Supplementary File S4. Level of expression of IFNγ, TNFα and GRZb in CD8^+^ TILs.** The level of expression was calculated as ΔMFI (the MFI measured in cytokine-positive CD8⁺ TILs minus the MFI calculated in the corresponding FMO control measured in the same population). Each dot indicates a single mouse.
